# *GJB2* and *GJB6* gene transcripts in the human cochlea: A study using RNAscope, confocal, and super-resolution structured illumination microscopy

**DOI:** 10.3389/fnmol.2022.973646

**Published:** 2022-09-20

**Authors:** Wei Liu, Helge Rask-Andersen

**Affiliations:** Department of Surgical Sciences, Head and Neck Surgery, Section of Otolaryngology, Uppsala University Hospital, Uppsala, Sweden

**Keywords:** human, cochlea, *GJB2*, *GJB6*, RNAscope, *in situ* hybridization

## Abstract

**Background:**

Gap junction (GJ) proteins, connexin26 and 30, are highly prevalent in the human cochlea (HC), where they are involved in transcellular signaling, metabolic supply, and fluid homeostasis. Their genes, *GJB2* and *GJB6*, are both located at the DFNB1 locus on chromosome 13q12. Mutations in *GJB2* may cause mild to profound non-syndromic deafness. Here, we analyzed for the first time the various expressions of *GJB2 and GJB6* gene transcripts in the different cell networks in the HC using the RNAscope technique.

**Materials and methods:**

Archival paraformaldehyde-fixed sections of surgically obtained HC were used to label single mRNA oligonucleotides using the sensitive multiplex RNAscope^®^ technique with fluorescent-tagged probes. Positive and negative controls also included the localization of *ATP1A1*, *ATP1A2*, and KCNJ10 gene transcripts in order to validate the specificity of labeling.

**Results:**

Confocal and super-resolution structured illumination microscopy (SR-SIM) detected single gene transcripts as brightly stained puncta. The *GJB2* and *GJB6* gene transcripts were distributed in the epithelial and connective tissue systems in all three cochlear turns. The largest number of *GJB2* and *GJB6* gene transcripts was in the outer sulcus, spiral ligament, and stria vascularis (SV). Oligonucleotides were present in the supporting cells of the organ of Corti (OC), spiral limbus fibrocytes, and the floor of the scala vestibuli. Multiplex gene data suggest that cells in the cochlear lateral wall contain either *GJB2* or *GJB6* gene transcripts or both. The *GJB6*, but not *GJB2*, gene transcripts were found in the intermediate cells but none were found in the marginal cells. There were no *GJB2* or *GJB6* gene transcripts found in the hair cells and only a few in the spiral ganglion cells.

**Conclusion:**

Both *GJB2* and *GJB6* mRNA gene transcripts were localized in cells in the adult HC using RNAscope^®^ *in situ* hybridization (ISH) and high resolution microscopy. Generally, *GJB6* dominated over *GJB2*, except in the basal cells. Results suggest that cells may contain either *GJB2* or *GJB6* gene transcripts or both. This may be consistent with specialized GJ plaques having separate channel permeability and gating properties. A reduction in the number of *GJB2* gene transcripts was found in the basal turn. Such information may be useful for future gene therapy.

## Introduction

Gap junctions (GJs) consist of paired protein hexamers forming direct bridges between cells. Such joined hemichannels or connexons may allow the transfer of electric impulses, small molecules, second messengers, nutrients, and microRNAs (miRNAs) between cells (Leybaert et al., 2003; Beltramello et al., 2004; Chang et al., 2008; Mammano, 2019). The gap junction networks are extensively developed in the epithelial and connective tissue systems in the mammalian cochlea (Kikuchi et al., 1995). Cx26 and Cx30 proteins are the dominating connexins and play essential roles in the hearing process in adults as well as during organ morphogenesis (Kikuchi et al., 1995; Lautermann et al., 1998; Xia et al., 1998; Jagger and Forge, 2015; Mammano and Bortolozzi, 2018; Mammano, 2019).

Mutations in the *GJB2* and *GJB6* genes that encode the Cx26 and Cx30 proteins are the most frequent causes of prelingual hereditary deafness of varying severity, and give rise to nearly half of the cases of autosomal recessive non-syndromic deafness (Kelsell et al., 1997; Cohn and Kelley, 1999; Grifa et al., 1999; Petit et al., 2001). Most of these mutations are present in the *GJB2* gene located in the *DFNB1* locus (Lerer et al., 2001; del Castillo et al., 2002; Pallares-Ruiz et al., 2002; Del Castillo et al., 2005; Boulay et al., 2013). The *GJB6* gene is positioned near the *GJB2* on chromosome 13. Moreover, immunostaining and co-immunoprecipitation indicate that Cx26 and Cx30 proteins commonly co-assemble, presumably into heteromeric or heterotypic molecular arrangements (Lautermann et al., 1998; Zhao and Santos-Sacchi, 2000; Ahmad et al., 2003; Forge et al., 2003; Sun et al., 2005; Zhao and Yu, 2006). Such molecular configurations could explain the human ear’s unique vulnerability to various genetic disruptions.

There is an active search for therapeutic options to restore auditory function that is disrupted by genetic connexin-related hearing loss. A major hurdle is the short and variable time frames for potential gene therapy due to the proteins’ essential role in early cochlear morphogenesis, their co-regulation, and inter-dependence. More knowledge is necessary regarding Cx26 and Cx30 gene expression, transcription, and protein composition within the composite cellular networks along the cochlear spiral. Assessment of the *GJB2* and *GJB6* gene expressions may improve our understanding of the function of different cellular networks in the human cochlea (HC) and how cell-specific changes and mutations may interact and cause Cx26/Cx30-based deafness. Recent human studies are partly contrasted with previous findings suggesting a separate expression of the two proteins in different domains, as revealed by super resolution structured illumination microscopy (SR-SIM) (Liu et al., 2016, 2017a).

Here, we strained to analyze *GJB2* and *GJB6* mRNA transcripts in the adult HC using RNAscope^®^ *in situ* hybridization (ISH) of archival paraformaldehyde-fixed sections. It was combined with immunohistochemical analyses of Cx26 and Cx30 proteins. In an earlier investigation, we analyzed Na/K-ATPase (NKA) gene transcripts—*ATP1A1*, *ATP1A3*, and *ATP1B1*—encoding the Na/K-ATPase α1, α3, and β1 isoforms together with *GJB6* and fractalkine gene transcripts (Liu et al., 2021a; Liu and Rask-Andersen, 2022). In this study, we extended these analyses to include *GJB2*, KCNJ10, and ATP1A2. KCNJ10, ATP1A1 and ATP1A2 were used as additional markers to accurately locate connexin gene transcripts in the stria vascularis cell components. A RNAscope^®^ employing the multiplex technique was used to examine whether *GJB2* and *GJB6* gene transcripts occur separately or in conjunction with each other in the cellular networks. Single mRNA gene transcripts appeared as bright foci, and results showed that the largest number of Cx26/Cx30 gene transcripts was in the outer sulcus epithelium, basal cells of the SV, and types I and II fibrocytes. A reduced number of *GJB2* gene transcripts were found in the basal turn. Multiplex gene data suggest that cells in the lateral wall may contain either *GJB2* or *GJB6* gene transcripts or both. These findings, together with protein localization, suggest that cells and GJ plaques may contain different channels with various levels of permeability and gating characteristics in the cochlear domains. The Cx26/Cx30 proteins may play a major role for K^+^ cycling along medial and lateral transcellular pathways. Further documentation of gene expression in the developing cochlea is warranted.

## Materials and methods

### RNAscope protocol

The technique used for the RNAscope^®^ analyses of HC sections was recently described (Liu and Rask-Andersen, 2022). In short, fixed-frozen human tissue sections underwent pretreatment with H_2_O_2_ (10 min, RT) and protease III (30 min, 40°C). After protease III incubation, the sections were exposed to a RNA-scope hybridization assay. Bio-Techne produced the probes. Hybridization was initiated by adding the RNA probe(s) (a mixture of probes termed C1, C2, and C3 channels) to the sections. Incubation was performed in a HybEZ™ Oven (Bio-Techne) for 2 h at 40°C. After hybridization, the slides were washed using 1x RNA-scope^®^ Wash Buffer. The sections were incubated with RNA-scope^®^ Multiplex FL v2 Amp 1, 2, and 3 (for 30 min/30 min/15 min, respectively) sequentially at 40°C to amplify the signal. For the RNA-scope^®^ Multiplex study, RNAscope^®^ Multiplex FL v2, HRP-C1, HRP-C2, and HRP-C3 were sequentially added to the sections (time 15 min). For signal detection, TSA-diluted Opal 520, 570, and 690 fluorophores were added to the sections after HRP-C1, C2, and C3, incubating the sections for 30 min each at 40°C. When the three Opal fluorophores were assigned to each channel, in present representation, two channels—C1 and C2—were assigned to *GJB2* and *GJB6*, KCNJ10 and GJB2, and Na/K-ATP1A1 and Na/K-ATP1A2 (Table 1). After each fluorophore incubation and rinse with 1x RNA-scope^®^ Wash Buffer, the RNA-scope^®^ Multiplex FL v2 HRP blocker was added and incubated in an oven for 15 min at 40°C. Then, sections were counterstained withxs 4’,6-diamidino-2-phenylindole (DAPI), and the slides were cover-slipped with ProLong^®^ Glass Antifade Mountant (Thermo Fisher Scientific, Waltham, MA, United States). The RNA-scope ISH produces a puncta of signals that represent a single mRNA transcript (Grabinski et al., 2015). The RNAscope^®^ Multiplex Fluorescent v2 assay allows simultaneous detection of up to four RNA targets (Wang et al., 2015). The standard RNAscope probes have 20 zz pairs with each consisting of 35-50 nucleotides and resulting in 1000bp coverage for any given transcript. Both the probes target different regions and they do not cross-detect each other. The GJB6 is targeting exon 5^1^ and GJB2 is targeting exon 2^2^.

**TABLE 1:** The mRNA probes used in the present investigation.

Gene	Species	Gene ID	Chromosome location	Cat#	Channel	Company
*GJB6*	h	10804	13q12.11	541391	C1	(b)
*ATP1A2*	h	477	1q23.2	568261	C3	b
*ATP1A1*	h	476	1p13.1	539891	C1	b
*GJB2*	h	2706	13q12.11	541381	C3	b
*KCNJ10*	h	3766	1q23.2	461091	C1	b

b; Bio-Techne.

### Semi-thin sectioning of surgically obtained human cochlea

**FIGURE 1:**
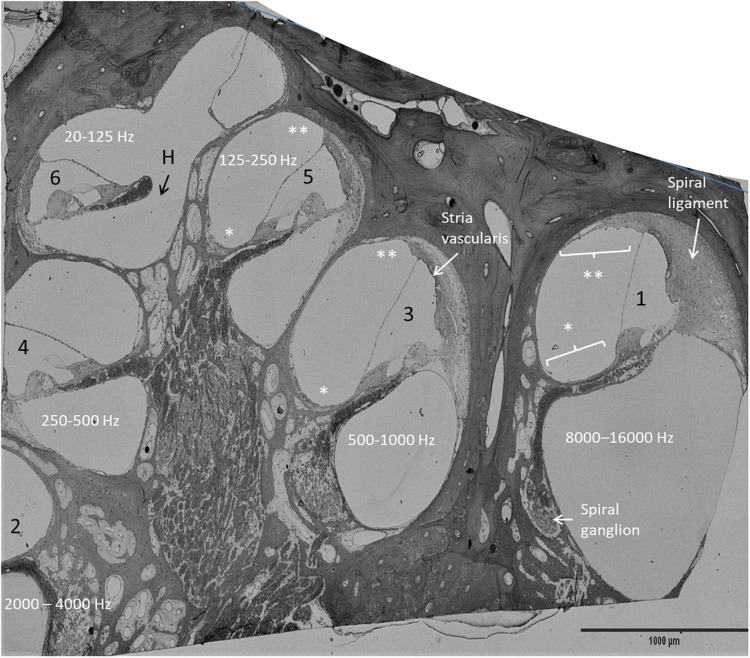
Mid-modiolar, semi-thin section of the right HC. Frequency distribution was estimated by assessing the number of cochlear turns. The anatomy of the OC and the lateral wall vary along the cochlear axis. The spiral ligament is more prominent in the high frequency region (1). *: mesothelium lining the floor of the scala vestibuli, ^**^: suprastrial fibrocytes, H, helicotrema.

Four archival human specimens were sectioned semi-thin, stained, photographed, and re-analyzed. The specimens were fixed in 3% glutaraldehyde and decalcified in 10% Na-EDTA. The bone was post-fixed in 1% osmic acid (OsO4, 75632 Sigma-Aldrich), and dehydrated and embedded in Epon (Resolution Performance Products, Houston, TX, United States). The technique was described earlier (Tylstedt et al., 1997). Photo images of various turns were examined in parallel with the mRNAscope^®^. Sections included different turns of the cochlea and were compared with the RNAscope findings at different regions (Figure 1).

### Immunohistochemistry

Techniques used for fixation, decalcification, embedment, and cryo-sectioning for immunohistochemistry were described in earlier publications (Liu et al., 2016, 2017a,b). Here, we used data obtained from studies on archival sections and previously obtained material. This included immune labeling of Na/K-ATPase (NKA) α1, α2, β1, NKCC, occludin, lamininβ2, Claudin-11, Cx30, and Cx26 (Table 1).

### Imaging and photography

The RNA-scope sections were analyzed using an inverted fluorescence microscope (Nikon TE2000; Nikon, Tokyo, Japan) and a three-filter digital camera (emission spectra maxima at 358, 461, and 555 nm). NIS Element BR-3.2 (Nikon) imaging software was used. Laser confocal microscopy was performed with a three-channel laser emission system. The software used was Nikon EZ-C1 (ver. 3.80), SR-SIM [Elyra S.1 SIM system with a 63x/1.4 oil Plan-Apochromat objective (Zeiss), sCMOS camera (PCO Edge), and ZEN 2012 software]. Multichannel SR-SIM imaging was performed using a laser and filter setup: 405 nm laser of excitation coupled with BP 420–480 + LP 750 filter, 488 nm laser of excitation with BP 495–550 + LP750 filter, 561 nm laser of excitation with BP 570–620 + LP 750 filter, and 647 nm laser of excitation with LP 655 filter. The resolution was measured with sub-resolution fluorescent beads (40 nm, Zeiss) in the green channel (BP 495–550 + LP750). An average point spread function (PSF) value was obtained from multiple beads using the built-in experimental PSF algorithm of the ZEN software. The resolution of the SR-SIM gave a lateral precision of 80 nm (Gustafsson et al., 2008). The volume resolution of three-dimensional (3-D) SIM is almost eightfold that of conventional microscopy (Schermelleh et al., 2008). The 3-D reconstructions and rendering of Cx26 (red) and Cx30 (green) protein expression were performed in spot detection mode using Imaris 9.5.1 (Bitplane, Zurich, Switzerland). The grid size of the frame on the 3-D images was 5μm. The number of *GJB2* gene puncta was counted using ImageJ particle analysis in the lateral wall at all three turns. Numbers were difficult to assess and had to be estimated in areas of transcription that burst in the cell nuclei. Both the total number of puncta and the number per cell (DAPI) were calculated and compared. Distortion of tissue may have influenced the results and focus remained primarily on obtaining comparative data of the three different turns.

## Results

### Immunohistochemistry and super resolution structured illumination microscopy

**FIGURE 2:**
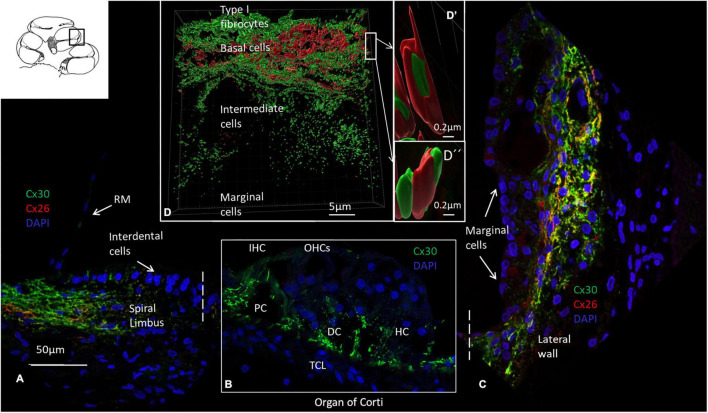
Confocal microscopy, SR-SIM, and Cx26 and Cx30 antibody labeling of the organ of Corti and lateral wall. **(A)** Cx26 and Cx30 are expressed in the spiral limbus fibrocytes. There is no expression in Reissner’s membrane (RM). **(B)** The OC expresses both Cx26 and Cx30, but in Figure B, only Cx30 is shown. IHC: inner hair cell. OHCs: outer hair cells. PC: pillar cell. HC, Hensen cell; DC, Deiters cell. TCL: tympanic covering layer. **(C)** Cx26 and Cx30 are both jointly and separately expressed in the lateral wall. Cx30 dominates except at the basal cell area of the stria vascularis. There is no expression in the marginal cells or in cells laterally margining the bone (type III fibrocytes). **(D)** SR-SIM using spot detection mode (Imaris 9.5.1) demonstrates separate and closely associated protein labeling with geometric arrangements in the lateral cochlear wall (D’ and D”). Cx30, but not Cx26, is expressed in the intermediate cells. The cochlear turn is not given.

An archival, semi-thin section of a decalcified HC fixed in 3% glutaraldehyde is demonstrated in Figure 1. This image shows the different anatomy of the OC and the lateral cochlear wall in the three turns. The spiral limbus and tectorial membrane are smaller and the spiral ligament is thicker against the basal high frequency region. Frequency mapping was estimated based on the number of cochlear turns. Confocal microscopy of Cx26 and Cx30 antibody co-labeling of the middle turn in an adult cochlea is shown in Figures 2A–C. Cx26 and Cx30 are both separately and co-expressed in the spiral limbus and lateral cochlear wall. Cx30 expression dominates in the OC (Figure 2B, not co-stained). SR-SIM using spot detection mode shows the distribution of Cx26 and Cx30 in the lateral wall (Figure 2D). There is a separate expression, with a band of Cx26 corresponding to the basal cell layer and the junction at the type I fibrocytes of the SV. Higher magnification confirms a close association between the two channel proteins (Figure 2D’,D”). The molecular arrangements of connexons (homomeric/heteromeric/heterotypic) cannot be assessed with certainty since the microscope resolution of 80 nm is far above the diameter of a Cx26 channel (Maeda et al., 2009). Cx30 is expressed solely in the intermediate cells, suggesting a homomeric molecular design. No GJ proteins were expressed in the marginal cells.

### RNAscope – confocal and super resolution structured illumination microscopy

**FIGURE 3:**
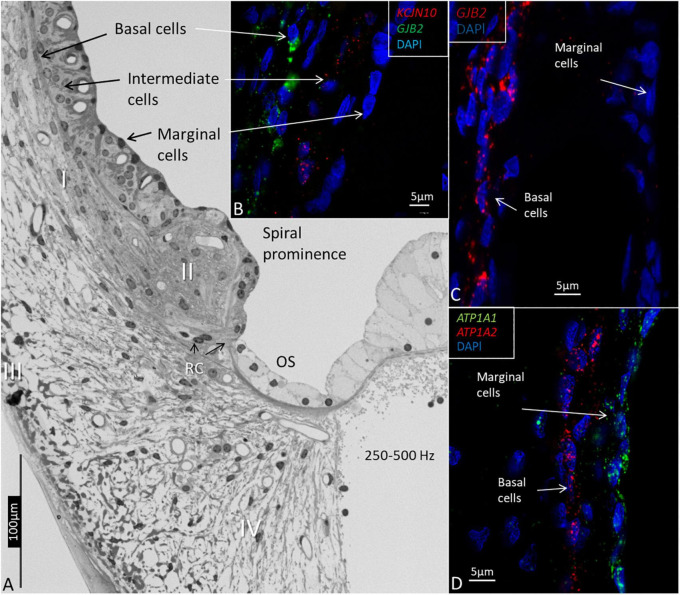
Light microscopy and confocal microscopy of the lateral wall of the upper-middle cochlear turn (frequency location 250–500Hz) showing marginal, intermediate, and basal cells of the stria vascularis. **(A)** The marginal cells are believed to be true epithelial cells, but there is no continuous basal lamina. Type I fibrocytes (I) of the spiral ligament lie close to the basal cells. The spiral prominence epithelium is flatter and is related to the type II fibrocytes. The outer sulcus (OS) epithelium, and Claudius and Hensen cells have a light cytoplasm and are fairly organelle-free. Root cells (RCs) project into the subepithelial space. Type III fibrocytes are located near the bone, and type IV faces the scala tympani. **(B)** RNAscope shows the distribution of *KCNJ10* and *GJB2* gene transcripts in intermediate and basal cells. **(C)** *GJB2* transcripts are located in the basal cells, but not in the marginal cells. **(D)** *ATP1A1* gene transcripts are seen in the marginal cells, while the basal cells contain *ATP1A2*.

The RNAscope^®^ and ISH identified the single *GJB2* and *GJB6* mRNA oligonucleotide transcripts in the formaldehyde-fixed sections of the HC. Decalcification maintained the cell integrity, and gene transcripts could be localized in the cell nuclei and cytoplasm. Na/K-ATPase and Kir4.1 gene transcripts (*ATPA1, ATP1A2*, and *KCNJ10*) were used as additional controls. *ATPA1* was only expressed in the marginal cells of the SV, while *KCNJ10* was expressed in the intermediate cells and *ATP1A2* in the basal cells (Figure 3).

**FIGURE 4:**
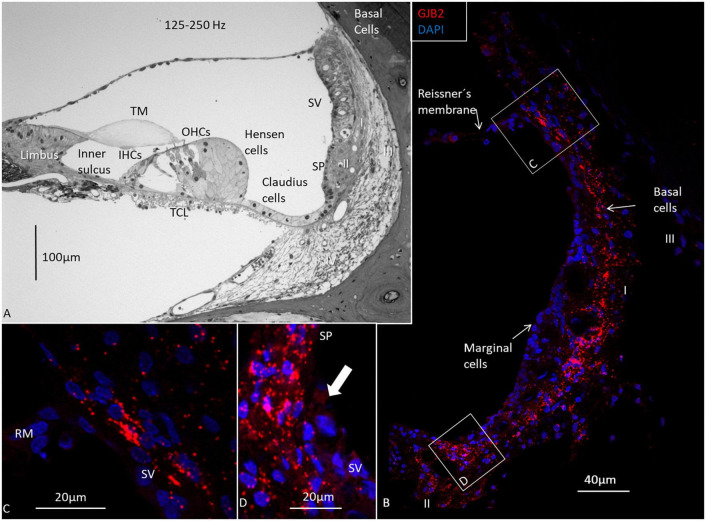
Light microscopy and confocal microscopy of the apical turn of the human cochlea at the frequency region 125–250 Hz. **(A)** A semi-thin section shows the stria vascularis (SV) and the relatively thin spiral ligament containing four different types of fibrocytes (I-IV). There is no suprastrial connective tissue layer containing type V fibrocytes. A thin layer of basal cells can be seen next to the type I fibrocytes. Type II fibrocytes lie beneath the spiral prominence (SP) epithelium. **(B)** Distribution of *GJB2* gene transcripts in the lateral wall. *GJB2* gene transcripts are concentrated at the basal cell (BC) region, while the marginal cells show no gene transcripts. A few red puncta can be observed at the intermediate cells. Gene transcripts are located in type I fibrocytes. Framed areas are shown in higher magnification in **(C,D)**. **(C)** Expression of *GJB2* at the angle between the Reissner’s membrane (RM) and SV. There are no transcripts in RM. **(D)** Enlargement of the angle between the SV with transcript-containing basal cells reaching the SP epithelium (arrow). IHCs, inner hair cells; OHCs, outer hair cells; TM, tectorial membrane; Limbus, spiral limbus.

**FIGURE 5:**
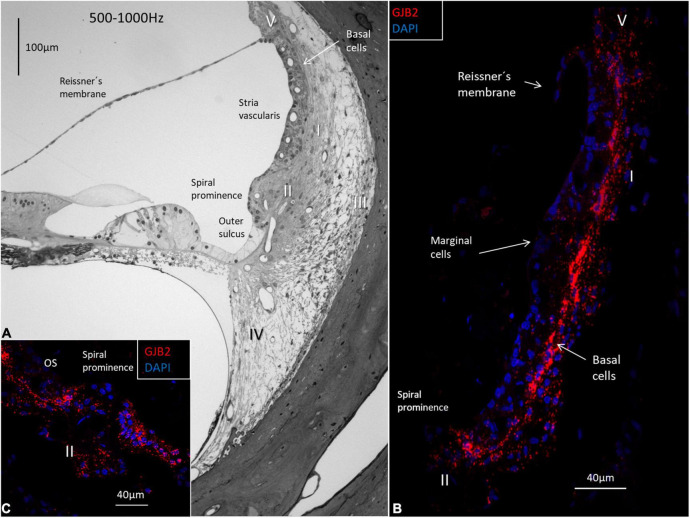
Light microscopy of the human OC and lateral wall at the frequency region 500-1000 Hz and confocal microscopy of *GJB2*. **(A)** The semi-thin section shows the stria vascularis and the spiral ligament with five different types of fibrocytes (I-V). Basal cells face the type I fibrocytes. The spiral prominence epithelium is thin and changes into a light epithelium in the outer sulcus (OS). **(B)** A large number of *GJB2* gene transcripts are concentrated in the basal cell region. There are no transcripts in the marginal cells. **(C)** There are few transcripts in the spiral prominence epithelium. A large number of transcripts are seen in the outer sulcus.

**FIGURE 6:**
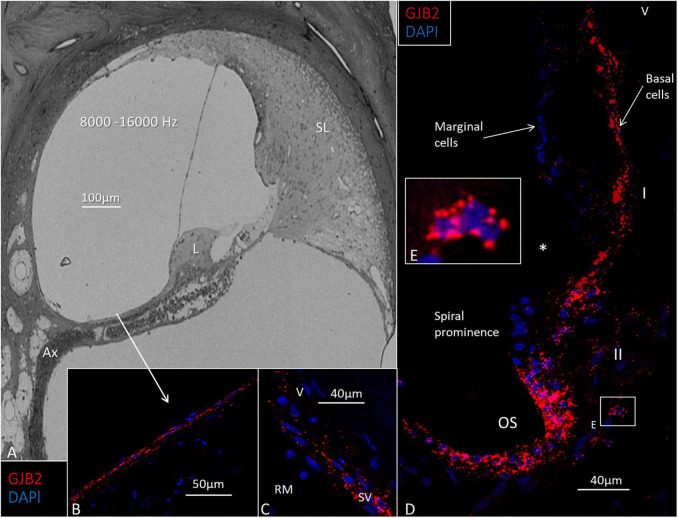
Light microscopy and *GJB2* in the basal turn of the cochlea at the high frequency region 8000–16000 Hz). **(A)** The spiral limbus (L) and tectorial membrane near the round window are smaller at this location, and the spiral ligament (SL) is more voluminous. Ax: peripheral axons. **(B)** *GJB2* gene transcripts in mesothelial cells lining the floor of the scala vestibuli. **(C)** *GJB2* gene transcripts at the lateral insertion of the Reissner’s membrane (RM). SV: stria vascularis. V: type V fibrocytes. **(D)** A large number of *GJB2* gene transcripts in the basal cells extend from the RM to the spiral prominence. *: disrupted marginal cells. The outer sulcus (OS) contains many *GJB2* gene transcripts. **(E)** Magnification of the framed area in **(D)** shows a type II fibrocyte rich in *GJB2* transcripts. I, II: fibrocytes types I and II.

**FIGURE 7:**
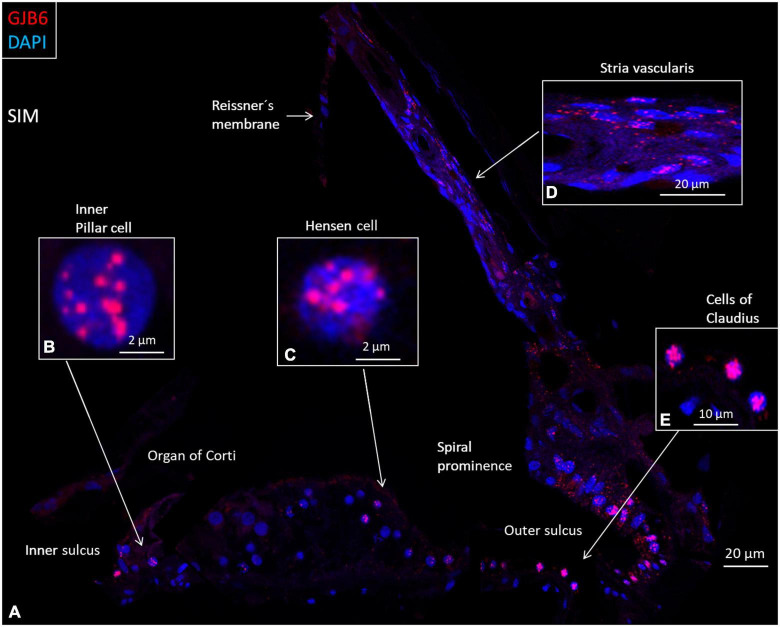
SR-SIM composite micrograph of *GJB6* gene transcripts in the basal turn of the human cochlea. **(A)** Many *GJB6* transcripts are seen in the outer sulcus and type I fibrocytes. Some Claudius cell nuclei contain large numbers of *GJB6* gene transcripts. A few supporting cells in the OC contain gene puncta. **(B)** Higher magnification of an inner pillar cell nucleus contains *GJB6* transcripts. **(C)** Higher magnification of a Hensen cell nucleus contains *GJB6* transcripts. **(D)** Higher magnification shows *GJB6* transcripts, mainly in the basal cells. **(E)** Claudius cells containing many gene transcripts. Cells in the tympanic covering layer contain no gene puncta.

**FIGURE 8:**
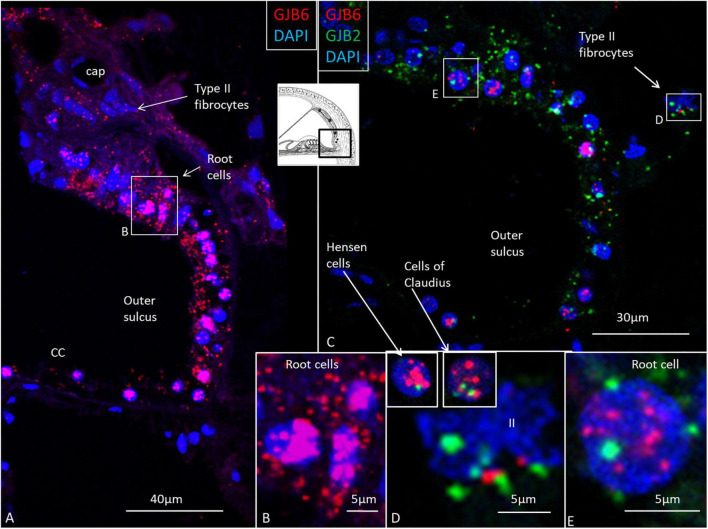
*GJB2* and *GJB6* gene transcripts in the outer sulcus in the basal turn of the cochlea. **(A)** Outer sulcus epithelium and root cells contain a large number of *GJB6* transcripts. Type II fibrocytes contain a moderate number of *GJB6* transcripts. The framed area is magnified in **(B)**. **(B)** Root cell nuclei are crowded with *GJB6* gene transcripts. **(C)** Multiplex RNAscope shows both *GJB2* and *GJB6* transcripts co-express in the outer sulcus epithelium. Framed areas are magnified in **(D,E)**, and show both gene transcripts present in a type II fibrocyte (II) and root cell nuclei. Insets in **(D)** show Hensen and Claudius’s cell nuclei expressing both transcripts, but with *GJB6* dominant. The diameter of the cell nuclei is around 5 microns.

The *GJB2* and *GJB6* gene transcripts were demonstrated in the cells of the spiral limbus, OC, and lateral wall in all three turns (Figures 4–8 and Table 2). The largest numbers of *GJB2* and *GJB6* puncta were detected in the outer sulcus epithelium, root cells, basal cells, and types II and I fibrocytes. In the SV, many *GJB2* and some *GJB6* gene transcript puncta were noted in the basal cells. Intermediate cells contained *GJB6*, but did not contain *GJB2* puncta. At the suprastrial region and the junction between the Reissner’s membrane and the SV, type V fibrocytes displayed both *GJB2* and *GJB6* transcripts. Type I fibrocytes contained a larger number of *GJB6* as well as *GJB2* gene transcripts, especially the cells located near the basal cells. While the upper and middle parts of the spiral prominence epithelium contained few *GJB2/GJB6* transcripts, the lower slope contained a large number of both *GJB2* and *GJB6* gene transcripts. The *GJB2*-containing basal cells merged with the spiral prominence epithelium and marginal cells in the apical turn (Figure 4D). A similar situation existed at the lateral insertion of the Reissner’s membrane (Figure 4C). A corresponding, but slightly different, anatomy was present in the middle and lower turns of the HC (Figures 5, 6).

**TABLE 2:** RNA gene transcripts.

	*GJB6*	*GJB2*
OSC	+ ++	++
HC	+ +	+
RC	+++	++
Type I	+ +	+
Type II	++	+
Type III	(−)	−
Type IV	+	+
Type V	+	+
MC	(−)	−
IC	+	−
BC	++	+++
SG(I)	(−)	(−)
SGC	(−)	(−)
SL	+ +	+
PC	++	?
BC	+ +	?
DC	++	?
HaC	−	−

OSC, outer sulcus cell; HC, Hensen cells; RC, root cell; MC, marginal cell; IC, intermediate cell; BC, basal cell; Type I–V fibrocytes; SG(I), spiral ganglion cell type I; SGC, satellite glia cell; SL, spiral limbus; PC, pillar cell; BC, border cell; HaC, hair cell; +, positive expression; −, no expression; (−), occasional puncta.

Relatively few gene transcripts were found in type IV fibrocytes, and none were detected in the type III fibrocytes. Type I and II fibrocytes, Hensen, Claudius, and outer sulcus cells contained many GJs on transmission electron microscopy (TEM). The spiral limbus contained many *GJB2* and *GJB6* gene transcripts, and many were contained in the cells lining the scala vestibuli floor (Figures 6A–C). A composite micrograph of the OC and lateral wall is shown in Figure 7. Claudius cell nuclei were crowded with *GJB6* gene transcripts, but there were only a few in the cytoplasm (Figure 7A). None were found in Reissner’s membrane or hair cells. In Deiters’, Claudius, and outer sulcus epithelial cells, the number of transcripts were also concentrated to the cell nuclei.

### *GJB6* dominates over *GJB2* except in the stria vascularis’s basal cells

**FIGURE 9:**
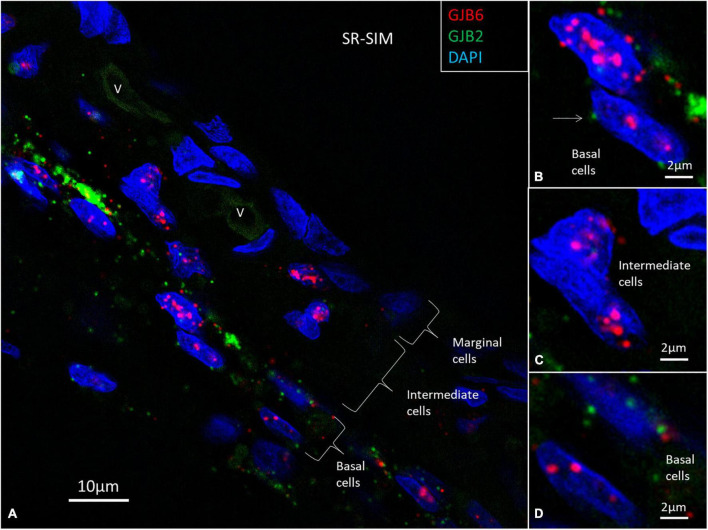
**(A)** SR-SIM and multiplex labeling of *GJB2* and *GJB6* transcripts in the human SV. **(B–D)** Intermediate cells solely contain *GJB6*, while basal cells contain either *GJB2* and *GJB6* transcripts separately or together in the same cell. This may suggest that GJ plaques consist of GJ channels with different properties and molecular arrangements. V: vessels. Arrow in B shows a perinuclear *GJB2* transcript.

Multiplex labeling of *GJB2* and *GJB6* transcripts in the HC shows that both transcripts are generally present in the epithelial and connective tissue cells (Figures 8, 9), except in the intermediate cells that only contained *GJB6* transcripts. An exceptional large number of *GJB2* and *GJB2* gene transcripts were noted in the outer sulcus and root cells. Both *GJB2* and *GJB6* were present in these cell nuclei (Figures 8B,D,E). Generally, the *GJB6* dominated over *GJB2* except in the basal cells where *GJB2* exceeded *GJB6* (Figure 9). We tried to assess whether cells may contain either *GJB2* or *GJB6* transcripts separately or if both are transcribed in the same cell by analyzing serial stacks of images. These results showed that some cell nuclei seemed to contain only one of the two transcripts while others contained both transcripts (Figures 9B–D).

### GJB2 gene transcripts in various cochlear turns

Results of ImageJ particle analysis of the number of GJB2 gene puncta in the lateral wall in the three turns are shown in Table 3. Results showed that there were fewer gene transcripts in the basal turn. This did not seem to result from a reduction of transcripts in each cell, but from a lower number of cells (DAPI) in the SV and spiral ligament at this level.

**TABLE 3:** Quantification of *GJB2* gene transcripts in the lateral wall of the three turns in a human cochlear section.

	Total number of gene transcripts	Total counted cells (DAPI)	Gene transcripts per cell
Base	1473	122	12.1
Middle	2250	210	10.7
Apical	2558	221	11.7

**FIGURE 10:**
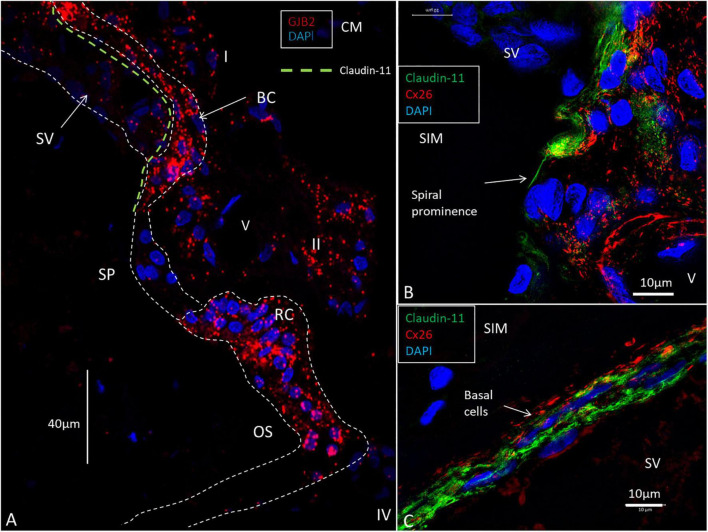
RNAscope of *GJB2* transcripts in the spiral prominence and outer sulcus. **(A)** The outer sulcus (OS), root cells (RC), and basal cells (BC) are rich in *GJB2* gene transcripts, while the spiral prominence epithelium virtually lacks gene puncta. The type I and II fibrocytes contain moderate numbers of gene transcripts. The green broken line shows the location of the Claudin-11 TJ barrier. **(B)** SR-SIM of Cx26 and Claudin-11 expression at the transitional zone between the stria vascularis (SV) and spiral prominence. A layer of the tight junction protein can extend to the spiral prominence (arrow). **(C)** SR-SIM of Cx26 and Claudin-11 protein expression in the basal cells of the SV. V: vessel. I: type I fibrocytes. II: type II fibrocytes. CM: Confocal microscopy. SIM: structured illumination microscopy.

To better understand the relationship between the *GJB2*-containing basal cells and the spiral prominence epithelium, we analyzed the co-expression of the tight junction (TJ) protein Claudin-11 and Cx26 (Figure 10A). It showed that basal cells jointly expressed TJ and GJ proteins and that TJ-expressing cells formed a thin layer that separated the basal and marginal cells from each other (Figures 10B,C).

**FIGURE 11:**
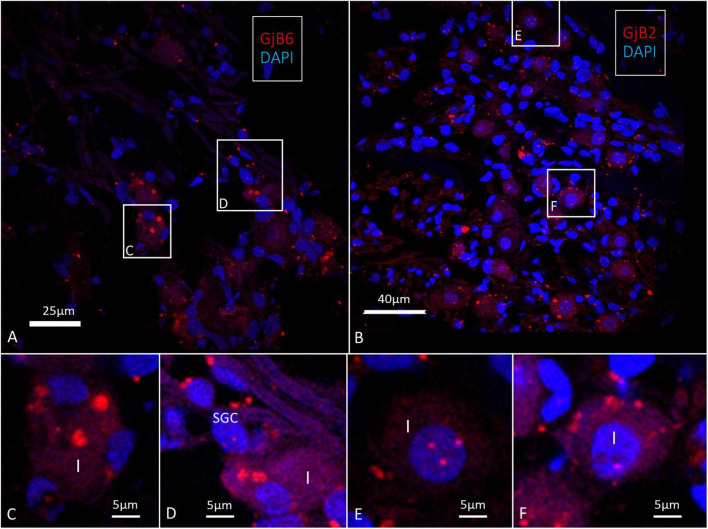
Confocal microscopy of *GJB2* and *GJB6* transcripts in the human spiral ganglion. **(A)** Occasional *GJB6* transcript puncta are seen in the type I ganglion cells and a few puncta can be seen in the surrounding cells, including the satellite glia cells (SGC). Framed areas are magnified in **(C,D)**. **(B)** A few *GJB2* gene transcript puncta are seen in the type I ganglion cell soma. Framed areas are shown in higher magnification in **(E,F)**. **(E)** Type I cell nucleus contains *GJB6* transcripts **(F)**. A type I cell contains *GJB6* puncta. Larger red-stained areas (non-puncta) are considered to be unspecific.

We found a few *GJB2* and *GJB6* transcript puncta present in the type I spiral ganglion cells as well as in the satellite glia cells (SGCs) (Figure 11). Larger stained areas were also seen in the peripheral cytoplasm of type I cells recognized as conceivably unspecific.

## Discussion

The RNAscope^®^ ISH of archival paraformaldehyde-fixed sections identified single *GJB2* and *GJB6* gene transcripts in the epithelial and connective tissue networks in surgically obtained HC using confocal microscopy and SR-SIM. A study limitation was the restricted amount of human tissue for more systematic gene localization. The results seem to be consistent with the distribution and expression of Cx26 and Cx30 isoforms described in recent investigations (Liu et al., 2017a,b).

The total number of *GJB2* gene transcripts was less in the base in accordance with the proteins in the guinea pig (Zhao and Yu, 2006). The specificity of the labeling was validated by using alternate gene biomarkers characterizing various cell domains in the SV. The *KCNJ10* encodes the inward rectifying potassium channel 4.1 (Kir4.1) that was specifically located in the intermediate cells (Wangemann et al., 1995; Hibino et al., 1997) while *ATP1A1* encoding Na/K-ATPase α1 was restricted to the marginal cells (Liu and Rask-Andersen, 2022). The basal cells expressed *ATP1A2* encoding Na/K-ATPase α2 and could be used to validate the various gene transcripts’ locations.

The GJs are composed of different connexins that post-translationally oligomerize into hexameric hemichannels before being inserted into the plasma membrane. They form direct intercellular passageways for transporting ions, ATP, glucose, miRNA, and second messengers (Dermietzel et al., 1990; Goodenough et al., 2003; Evans and Martin, 2009; Zhu et al., 2015; Laird and Lampe, 2018). Several mutations affecting the *GJB2* gene are known to give rise to hearing loss of varying severities, and there is an active search for therapeutic options. Deafness-causing mutations in the *GJB2* gene may give rise to both non-syndromic and syndromic deafness, but are mostly autosomal recessive point mutations (Wang et al., 2003). Large deletions in the DFNB1 locus may also affect *GJB2* transcription and regulatory sequences as well as *GJB6*, and result in profound hearing loss (Del Castillo and Del Castillo, 2017). The molecular and structural arrangements of GJs may affect phenotypic expression and the consequences of different mutations.

Our knowledge of the structure of cochlear GJs is mostly based on animal models, which have suggested that Cx26 and Cx30 generally co-assemble, particularly in mesenchymal fibrocytes where they are believed to form unique heteromeric or heterotypic assemblies (Lautermann et al., 1998; Zhao and Santos-Sacchi, 2000; Forge et al., 2003; Sun et al., 2005). The GJ channels containing multiple connexins could have separate gating properties that contribute to functional diversity in intercellular communication (Sosinsky and Caspar, 1995; White and Bruzzone, 1996). Co-immunostaining and co-immunoprecipitation have indicated that proteins could even co-assemble in the same plaque (Ahmad et al., 2003). Such arrangements could enhance intercellular Ca^2+^ signaling that is essential for inner ear homeostasis and explains the ear’s vulnerability to connexin derangements.

### Hybrid plaques

There is uncertainty of whether human GJ channels in the OC and lateral wall of the cochlea are organized as heteromeric or homomeric connexons. Recent studies submit that Cx26 and Cx30 can be separately expressed, forming closely associated GJ plaques with Cx26 subdomains that occasionally appear as “hybrid plaques” (Liu et al., 2016, 2017a). Different GJ channels could populate the same GJ plaque or closely interact with different plaques. This was determined by freeze-fracture immunogold labeling (Fujimoto, 1995) and astrocytes expressing Cx26, Cx30, and Cx43 in rats (Rash et al., 2001). The present study also supports this arrangement. A high degree of complexity of the transcriptional co-regulation mechanism may be present in these sophisticated human cellular networks (Kim et al., 2012). SR-SIM showed that both *GJB2* and *GJB6* gene transcripts were separately localized in different cells, but could also appear in the same cell. Cx26 and *GJB2* gene transcripts were enriched in the basal cell layer, which forms a critical stratum for generating the endo-cochlear potential (EP) by passive movement of potassium ions across its apical membranes (Tasaki and Spyropoulos, 1959; Salt et al., 1987). Cx26 and Cx30 co-staining showed a distinct layer of Cx26-positive cells with less Cx30 (Figure 2). The marginal cells contained neither *GJB2* nor *GJB6* gene transcripts. The basal cells may comprise preferentially homomeric or homotypic Cx26 and Cx30 hemichannels, while a heterotypic molecular arrangement seems less likely considering the IHC and *GJB2* and *GJB6* transcript distribution. It may favor the presence of molecularly separate GJs and possibly hybrid junctions with plaques consisting of both Cx26 and Cx30 channels (Liu et al., 2017a,b). They may have different properties and act synergistically, including the generation of the EP, a prerequisite for human hearing. The rich content of the Cx26 protein is reflected by the large number of *GJB2* gene transcripts and could clarify the major effects caused by *GJB2* gene disruption on human hearing and EP that is not readily compensated for by Cx30. Cochlear cell degeneration of cCx26 null mice was more extensive and rapid than in Cx30 null mice (Sun et al., 2009). One would expect that genetic deletion of one of the genes would result in a similar pathology if it was heterotypically arranged. The dominating effect of Cx26 was also shown by the fact that hearing can be normal without Cx30 in cases where Cx26 levels are preserved (Ahmad et al., 2007; Boulay et al., 2013; Jagger and Forge, 2015).

### Hemichannels

Notably, intermediate cells only contained *GJB6* gene transcripts and Cx30 protein. Transcripts encircled the blood vessels, which is consistent with murine studies showing that Cx30, but not Cx26, is present in the SV’s intermediate cells (Cohen-Salmon et al., 2002). They found that, in homozygous Cx30 mutations, a down-regulation of betaine homocysteine S-methyltransferase in the stria capillaries resulted in endothelial dysfunction and hearing loss. It is not known whether these channels represent GJs or hemichannels that form routes between the extracellular space and cytosol. Such channels have been implicated in paracrine signaling through plasma membrane release of various messengers, such as ATP and glutamate, nicotinamide adenine dinucleotide, and prostaglandins (Wang et al., 2013). They may also be involved in regulating inflammatory responses (Baroja-Mazo et al., 2013; Willebrords et al., 2016).

### Epithelial *GJB2* and *GJB6* and K^+^ recirculation

Supporting cells in the OC, including Hensen and Claudius cells, contain a moderate number of *GJB2* and *GJB6* gene transcripts, mostly located in the same cell nuclei. The precise number was difficult to assess since fluorescent puncta coalesced into larger assemblies, possibly representing transcriptional bursts. No transcripts were found in the hair cells or in the cells of the tympanic covering layer (TCL) located beneath the basilar membrane. The large number of transcripts in the outer sulcus epithelium, root cells, type I and II fibrocytes, and proteins may support the view that there is a lateral flow of K^+^ ions from the hair cell region to the lateral wall and back to the SV. Many cells contained a large number of transcripts, both in the cell nuclei and cytoplasm, while others contained a lower number. This suggests that cells show a variable transcription activity. SR-SIM and Cx30 immune staining indicated that open hemichannels are also present in the supporting cells in the human OC (Liu et al., 2017a). Inner and outer pillars and Deiters’ cells face cortilymph, and hemichannels could mediate autocrine/paracrine signaling by releasing ATP (Leybaert et al., 2003). They could also play an important role in maintaining low extracellular glutamate levels in order to avoid neuronal death from glutamate excitotoxicity and thereby maintain synaptic transmission (Ye et al., 2003). Cx30 greatly dominates Cx26 in the adult human OC.

### *GJB2* and *GJB6* in the scala vestibuli

*GJB2* and *GJB6* gene transcripts were also detected in the mesothelial lining of the floor of the scala vestibule and supra-limbus cells. These cells expressed Cx26 and Cx30 (Liu et al., 2016), suggesting that both proteins have the capacity to trap the sequestered K^+^ ions and bring them to the suprastrial fibrocytes in the lateral wall. This could be an additional route for recirculating K^+^ from the inner hair cell region, inner sulcus, and spiral limbus contributing to endolymph production from perilymph (Sterkers et al., 1982; Marcus et al., 2002; Konishi et al., 1978). The GJs were shown in both the supralimbal and mesenchymal cells lining the scala vestibuli in the rat (Kikuchi et al., 1995). More studies are needed in order to verify whether GJs are present between these cells in the HC.

### *GJB2* and *GJB6* gene transcripts and the spiral prominence

Researchers resembled the basal cell layer with the placenta barrier, where both GJs and TJs regulate the exchange of fluids and small metabolites between blood supplies (Metz et al., 1978; Kikuchi et al., 1995). A breach of this barrier could be detrimental, and a reciprocal regulation between GJ and TJ proteins was suggested (Giepmans and Moolenaar, 1998; Kojima et al., 2002). Gap junctions were shown to influence TJ permeability, such as Claudin and Occludin which also co-immuno-precipitate (Kojima et al., 2001; Dbouk et al., 2009). A similar interaction between GJ and TJ may occur in the cellular networks of the SV. Claudin-11 was shown to be indispensable for hearing and for maintaining the EP, but not of a high K^+^ concentration in the endolymph (Kitajiri et al., 2004a,b). Human studies show that Cx26 partly co-assembles with the TJ Claudin-11 along the basal cell layer. Unexpectedly, *GJB2*-containing basal cells reached the upper slope of the spiral prominence epithelium and seemed to face endolymph. Staining showed that Claudin-11 insulates basal cells from spiral prominence and endolymph (Liu et al., 2017b) (Figures 10A–C). The TJ protein forms laminar insulations between the *GJB2*-positive basal cells and spiral prominence epithelium (Figure 10). A similar arrangement exists at the insertion of Reissner’s membrane. This compartmentalization could be pivotal to separating the spiral prominence from the SV in order to polarize K^+^ flow into it. The spiral prominence may be critical as a circuit for ions entering and leaving this region.

### Few GJB2 and GJB6 transcripts in the human spiral ganglion

There were few *GJB2* and *GJB6* gene transcripts puncta that were detectable in the spiral ganglion (Figure 11). This is surprising since IHC showed positive staining of both proteins (Liu et al., 2009, 2019, 2021b). Cx30 was localized in discrete deposits in type I spiral ganglion cell bodies while Cx26 was more diffusely stained. Differences may depend on the antibodies that were used. In an earlier study, larger areas were found with assumed *GJB6* expression (Liu et al., 2021b). A similar pattern was noted in the present study, but with few transcript puncta present in type I spiral ganglion cells and satellite cells (Figure 11). Further studies using additional molecular techniques are necessary in order to establish the degree to which *GJB2* and *GJB6* transcripts are present in the human spiral ganglion cells. There was no positive Cx30 staining after double-labeling with TUJ1 or in parvalbumin-positive nerve endings beneath the hair cells. This suggests that Cx30 may not be involved in nerve excitation in the human OC. Cx36 is the principal neuronal connexin in the mammalian CNS, but the Cx36 gene transcript, GJD1, was not detected in an earlier investigation (Liu et al., 2021b).

### *GJB2* and *GJB6* transcription rates

A noteworthy number of *GJB2* and *GJB6* gene transcripts in outer sulcus and spiral ligament cells may suggest a high transcription rate. The GJ connexins have been shown to have a remarkably short half-life of just a few hours (Laird, 2006). Connexin proteins are continually synthesized and degraded depending on physiological demands, and genes may be upregulated with increased protein levels and altered localization (Risek et al., 1990). Gap junctions can remodel, such as in cardiac disease where Cx43 serves to electrically convey impulses between myocytes (Dhein and Salameh, 2021). Connexin channels may also change between an active and inactive state, regulated by phosphorylation processes (Bukauskas et al., 2000). A high turnover rate indicates that there is a rapid synthesis and replenishment essential to upholding function. Cell-cell coupling was previously considered stationary, but it is now realized that gene expression and transcription can be highly irregular, with spontaneous variability occurring in some cells due to environmental conditions. Such “burst” transcription has been described in several mammalian tissues (Bahar Halpern et al., 2015). Insults may remodel GJ coupling with rapid degradation in the plasma membrane, removal, and turnover, which are critical for function. Noise- and age-dependent hearing loss have been associated with Cx26 dysregulation (Wang et al., 2014; Wu et al., 2014; Zhou et al., 2016; Fetoni et al., 2018). We found a reduction in the number of expressed genes in the basal turn (Table 3), supporting an earlier finding of diminished Cx26 and Cx30 activity at the high-frequency level (Zhao and Yu, 2006). Further elucidation is also need on whether this relates to age-related hearing loss. Inflammatory changes in spiral ligament cells could also interrupt GJ K^+^ recycling and cause sensorineural hearing loss. Methods to pharmacologically target and modify GJ trafficking have been suggested as a way to restore altered coupling and transcellular electrical activity in other organs. Whether such intriguing strategies could also be applied to the inner ear to treat hearing impairment remains to be determined.

## Conclusion

**FIGURE 12:**
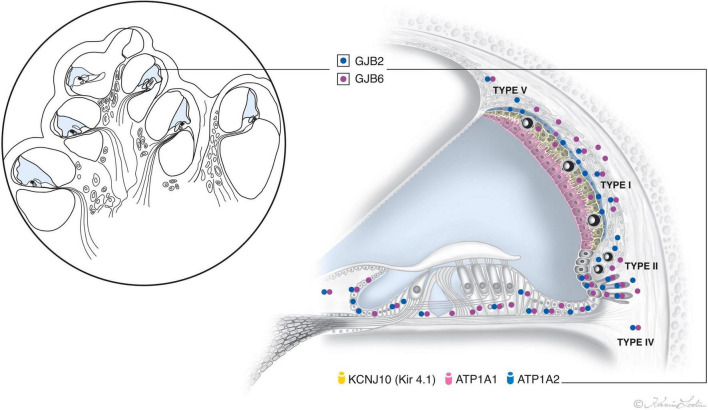
Illustration showing the relative distribution of *GJB2* and *GJB6* gene transcript puncta in the epithelial and connective tissue networks in the human cochlea. The location and number of transcripts vary among cells in the lateral wall, with the largest number appearing in the outer sulcus cells, root cells, and types I and II fibrocytes and basal cells. *GJB2* dominates in the basal cell layer. The intermediate cells only express *GJB6*. No *GJB2* and *GJB6* gene transcripts are expressed in the marginal and sensory hair cells. Two GJ systems exist in the HC – Epithelial and connective tissue. The epithelial system consists of interdental cells of the spiral limbus, supporting cells of the organ of Corti (OC), bordering cells, phalangeal cells, Hensen cells, Deiters’ cells, inner and outer sulcus cells with root cells projecting into the spiral ligament, and marginal cells of the stria vascularis. The connective tissue system consists of fibrocytes of the spiral limbus, lateral wall including the basal and intermediate cells of the stria vascularis (SV), suprastrial fibrocytes, and mesenchymal cells lining the scala vestibuli and supralimbal cells (Kikuchi et al., 1995).

The present study shows for the first time the combined distribution of *GJB2* and *GJB6* gene transcripts in various turns of the HC using RNAscope technique (Figure 12). The transcripts are often co-localized, but were also separately expressed with a heterogeneous localization in the stria vascularis. The *GJB6* transcripts, but not the *GJB2*, were found in the intermediate cells while the *GJB2* transcripts dominated over *GJB6* in the basal cells. The largest amount of gene transcripts were found in the outer sulcus epithelium, type I and II fibrocytes, and basal cells. A reduction in the number of *GJB2* gene transcripts were noted in the basal turn. There were no gene transcripts in the sensory cells or Reissner’s membrane. Few *GJB2* and *GJB6* gene transcripts were distinguished in the spiral ganglion. The findings suggest that cells and GJ plaques may be specialized and contain different channels with various permeability and gating characteristics in the cochlear domains.

## Data availability statement

The original contributions presented in this study are included in the article/supplementary material, further inquiries can be directed to the corresponding author.

## Ethics statement

Human cochlea tissue used in prior studies was reused, semi-thin sectioned, and photographed (Tylstedt et al., 1997). The study was approved by the Local Ethics Committee (no. 99398, 22/9 1999, cont., 2003, no. C254/4; no. C45/7 2007, Dnr. 2013/190). The study adhered to the rules of the Declaration of Helsinki. Specimens were earlier obtained from patients suffering from life-threatening petroclival meningioma undergoing transcochlear surgery. The patients/participants provided written informed consent to participate in this study.

## Author contributions

WL performed IHC and RNAscope. HR-A and WL documented the results, interpreted data, and wrote the manuscript. Both authors contributed to the article and approved the submitted version.

## Footnotes

1
https://www.ncbi.nlm.nih.gov/nuccore/NM_001110219.2

2
https://www.ncbi.nlm.nih.gov/nuccore/NM_004004.5
